# “Stuck Due to COVID”: Applying the Power and Control Model to Migrant and Refugee Women’s Experiences of Family Domestic Violence in the Context of the COVID-19 Pandemic

**DOI:** 10.3390/ijerph22040627

**Published:** 2025-04-16

**Authors:** Azriel Lo, Georgia Griffin, Hana Byambadash, Erin Mitchell, Jaya A. R. Dantas

**Affiliations:** Curtin School of Population Health, Curtin University, Bentley 6102, Australia; azriel.lo@curtin.edu.au (A.L.); georgia.griffin@curtin.edu.au (G.G.); tserenkhand.byambadash@curtin.edu.au (H.B.); erin.mitchell@curtin.edu.au (E.M.)

**Keywords:** domestic violence, migrant, refugee, women, gender, COVID-19, pandemic, gender-based violence

## Abstract

The COVID-19 pandemic had acute and lasting gendered impacts around the world, with UN Women declaring a shadow pandemic of violence against women. This study aimed to explore the impact of the COVID-19 pandemic on migrant and refugee women’s experiences of family domestic violence (FDV) in Western Australia (WA) using a community-based participatory research approach. Thirty-eight interviews and two qualitative surveys conducted with 27 women were included in the analysis. Interview and survey data underwent reflexive thematic analysis informed by the Power and Control Wheel, generating three themes and ten subthemes: (1) the facets of violence women experienced (isolation; economic violence; emotional violence; visa vulnerabilities; fear and uncertainty), (2) the systemic enablers of FDV and barriers to seeking help (FDV service provision; the immigration system), and (3) the impact of the COVID-19 pandemic and government measures on women and family (enabling FDV; reducing the impact of FDV; COVID-19 paled into insignificance). While for some migrant and refugee women, government measures facilitated access to support for FDV, our findings show that for others, the COVID-19 pandemic exacerbated and became part of the violence they had already experienced. Recommendations for tailored FDV and other support during the COVID-19 pandemic are discussed, including the expansion of family violence provisions for all visa types.

## 1. Introduction

### 1.1. COVID-19 and Global Impacts

In March 2020, the World Health Organization (WHO) declared a pandemic in response to the spread of a novel coronavirus, COVID-19 [1]. The emergence and rapid spread of this virus had an acute impact, with 772,386,069 confirmed cases and 6,987,222 deaths reported to the WHO as of 13 December 2023 [2]. Global excess mortality between 1 January 2020 and 31 December 2021 alone was estimated at 14.73 million deaths [1]. Evidence soon emerged that those who had lived through mild to severe COVID-19 infection could also go on to develop a range of physical and psychological symptoms, termed long COVID [3].

The COVID-19 pandemic placed an increased and, in some countries, overwhelming burden on healthcare systems [4]. Governments implemented varied emergency public health responses, including containment measures such as quarantines, curfews, and lockdown measures [4], placing approximately 3.9 billion people in isolation by April 2020 [4]. These measures, along with the virus itself, have had lasting socioeconomic, health, and gendered impacts [5].

### 1.2. COVID-19 and Lockdowns in Australia

Despite Australia’s geographic isolation [6], COVID-19 was first recorded in the country on 25 January 2020 [4] and in Western Australia (WA) on 21 February 2020 [7]. In March 2020, a State of Emergency was declared in WA, following which a range of public health responses were implemented. These included lockdown measures, social isolation and distancing measures, enforced quarantine, the closure of state and national borders, and limitations to welfare and health service access [6,7,8]. Social venues and religious and cultural institutions were also closed [9]. From May 2020, restrictions were alternately eased or reinstituted in response to the level of the perceived threat of COVID-19 outside of and within WA [9]. The easing of restrictions allowed WA residents to resume their daily activities (e.g., attending school or work) with selected measures still in place (e.g., social distancing or mask wearing). The WA state government ended the official State of Emergency and the Public Health State of Emergency associated with the COVID-19 pandemic on 4 November 2022 [10].

Government public health responses affected different individuals in different ways [10]. In the WA context, for some, no changes or positive changes in health behaviors were reported during lockdown measures, whereas for others, poor mental health symptoms and negative health behavior changes, such as increased alcohol intake, loneliness, and psychological distress, were reported [11]. Public health measures compounded pre-existing issues of unemployment, financial stress, and support service availability and accessibility for the WA population broadly, and particularly for culturally and linguistically diverse (CaLD) communities [8]. In Australia, the descriptor CaLD is used in policy and research to reflect the multiculturalism and diversity of languages spoken by individuals and within some communities residing in Australia [12]. In 2024, the COVID-19 Response Inquiry Panel released a report that provided guiding recommendations and actions for change to enhance Australia’s responses to public health emergencies in the future, recommending more bespoke interventions informed by the experiences of CaLD communities to address health, social, and economic disparities faced by these communities during public health emergencies [13].

### 1.3. COVID-19 and Family Domestic Violence

Both the spread of COVID-19 and public policy responses had gendered impacts on those already considered vulnerable due to pre-existing societal and systemic inequities [14,15]. The nature of lockdown measures, in which the public was required to stay at home and access to social supports and health services was restricted, exacerbated the violence experienced by women who were not safe at home due to pre-existing family domestic violence (FDV) and limited their ability to seek help. FDV refers to behaviors that cause, or intend to cause, fear and/or harm within intimate partner and family relationships [16]. FDV is not limited to physical harm but encompasses coercive control and emotional and sexual abuse [16]. It can be perpetrated against anyone of any gender; however, the focus of this study is on FDV perpetrated against women. In 2020, UN Women announced a shadow pandemic of violence against women as part of a global public awareness campaign to recognize and mitigate the exacerbation of FDV during the COVID-19 pandemic, highlighting the severity of FDV in the midst of the pandemic and the need for timely interventions [17,18].

Gender-based violence, particularly against migrant women, is known to increase in prevalence following disasters, such as conflicts and pandemics [19]. Legal status, poverty, pre-existing health issues, limited agency, gender inequality, and language and cultural barriers have been identified as intersectional factors increasing migrant women’s vulnerability to gender-based violence, along with other barriers such as poor access to care [19]. The role of structural violence, that is, harm experienced due to cumulative and interacting structural inequities, has also been highlighted as exacerbating pre-existing vulnerabilities faced by forced migrant women who have survived sexual and gender-based violence, both in Australia and globally, during the COVID-19 pandemic [20].

### 1.4. FDV in Australia

In Australia, not only did reports of FDV increase in frequency and severity during the COVID-19 pandemic, but there was also an increase in the reporting of incidents of FDV occurring for the first time [14]. Policies implemented in Australia during the pandemic weakened reproductive rights, restricted prevention and support services, and reinforced gender inequalities [15].

Evidence centering the experiences and voices of victim-survivors is currently inadequate and is needed to inform the design, implementation, and evaluation of new approaches to respond to intimate partner violence [14]. The Australian National Research Agenda to End Violence Against Women and Children 2023–2028 clearly sets out a call for more research addressing violence at societal and systemic levels, stating that “to address structural inequities, services and systems need to respond to, and create space for, the voices of people most at risk of marginalization” [21] (p. 15). This study offers such key insights into the FDV experiences of CaLD migrant and refugee women, revealing not only the impacts of the COVID-19 pandemic but also clear and persistent systemic inequities that must be addressed.

## 2. Materials and Methods

### 2.1. Aim

This article reports on qualitative data collected as part of the broader mixed methods multi-phased SHAKTI project. The findings reported in this article aim to explore the impact of the COVID-19 pandemic on CaLD migrant and refugee women’s experiences of FDV.

### 2.2. Study Context

The larger SHAKTI project uses a community-based participatory research (CBPR) approach to document the gendered impacts of the COVID-19 pandemic and the associated isolation measures on CaLD migrant and refugee women who were experiencing or had experienced FDV to facilitate the co-design of policy and programmatic recommendations and a frontline intervention. This paper focuses on reporting the results from phase one of this multi-phase research project. Women recruited in this phase will be invited to participate in subsequent phases of this project (i.e., intervention evaluation).

This study was conducted in Perth, WA, the most geographically isolated capital city in Australia [22]. Perth is a sprawling metropolis of approximately 2.1 million people [23,24], of which only 59.5% were born in Australia. Common languages other than English spoken at home include Mandarin, Italian, Vietnamese, Punjabi, and Cantonese [24], offering insights into the increasingly multicultural makeup of Perth.

### 2.3. Community-Based Participatory Research Approach

This research is underpinned by a CBPR methodology. Community strengths are valued within this collaborative approach to address health disparities and enhance the effectiveness and sustainability of health promotion initiatives [25,26]. Informed by CBPR, women, key community and service informants, and researchers formed a partnership to address FDV as experienced by CaLD migrant and refugee women [23,27]. A key aspect of this partnership was the formation and ongoing engagement of a Consumer Advisory Group (CAG). The CAG guides the broader SHAKTI project, providing feedback and expert insight on the research design, implementation, and findings. It consists of women with FDV lived experience, FDV service providers (e.g., social workers, advocates, and team leaders), FDV researchers, and general community members. To facilitate participant recruitment, key frontline agencies were identified as research partners with the assistance of the CAG. These agencies also offered safe spaces for data collection where appropriate. CBPR principles were also implemented during data analysis, guiding the research team to interpret the data whilst considering the consumer (i.e., lived experience of FDV CaLD migrant and refugee women) benefits to ensure that their needs were advocated and that partner organizations received evidence-based resources to support this population [23,27]. The CAG continued to have an active role in the data analysis, facilitating the interpretation of the research findings and the co-design of the consequent phases of the broader SHAKTI project. 

### 2.4. Participant Recruitment

Participants were recruited through snowball and purposive sampling approaches [28]. Five community and frontline organizations partnered with the research team, in alignment with the CBPR methodology [23]. Representatives from these organizations offered women who met the eligibility criteria information about the research. Inclusion criteria included identifying as a woman from a CaLD refugee or migrant background who experienced FDV or the ongoing impacts of FDV during and before the COVID-19 pandemic, being over 18 years of age, and having basic English language proficiency. The main indicators used in this study to define CaLD status were country of birth and ethnicity. If a woman expressed interest in participating, they were contacted by a member of the research team. This sampling approach was chosen as it enabled the research team to recruit women who met the inclusion criteria whilst ensuring that the women were not at immediate risk where this research would jeopardize their safety. Of the 41 eligible women who expressed interest through partner organizations, 27 women participated in the interview(s) or qualitative survey.

### 2.5. Data Collection

Data were collected through narrative and/or semi-structured or condensed interviews and qualitative surveys inclusively from February 2023 to May 2024. In total, 38 interviews and two qualitative surveys were conducted with a total of 27 eligible women. Interviews were conducted individually by one of three authors (GG, HB, or AL). Data were collected in a private setting that was identified as safe by each woman, such as an office at the partner organization they were familiar with, a room/an office at the university campus, or by telephone or Microsoft Teams. Aligned with the CBPR methodology, data collection was responsive to participant and CAG feedback, with multiple data collection tools used to enable women to share their experiences in a way that felt safe to them. For example, following feedback that some women had expressed interest in participating but were not comfortable with the interview format, a qualitative survey was developed based on the prior interview guides. The qualitative survey consisted of 19 open-ended questions selected from the narrative and semi-structured interviews. Questions included, *how did the pandemic affect your experience of domestic violence or the ongoing effects of domestic violence?* The qualitative survey was available in English only, hosted in Qualtrics [29], and a link to access the survey was sent directly to women who requested it. Two women completed the qualitative survey. Most women chose to participate in a narrative and/or semi-structured or condensed interview conducted in English. A female interpreter located in another state was arranged to interpret by telephone for interviews with three women at their request. For one woman, a support person was present to assist in interpreting.

All interview schedules were created by the research team and finalized in consultation with the CAG. The narrative interview guide comprised nine open-ended questions designed to invite the participant to respond in narrative form, eliciting their experiences in the context of their life histories [30]. Questions included, *would you mind telling me about your experience of the COVID-19 pandemic* or *would you mind telling me about any strategies you find helpful to overcome or manage the ongoing effects of domestic violence*? The semi-structured interview guide comprised 54 questions designed to draw out more focused reflections on how the women navigated the complex intricacies of their lives, and the facilitators and barriers to doing so [31]. Questions included, *‘what were the main changes to your daily life during the COVID-19 pandemic?’* or *‘what were the effects for you (and your children) of these changes during this time?’*. In response to participant feedback, the narrative and semi-structured interview guides were condensed midway through the data collection period into a single interview guide to minimize the burden of participation. The condensed interview guide comprised 46 questions selected from the narrative and semi-structured interviews. Prompts, such as *‘can you tell me more about that?’*, were used to establish clarifying details. For the first 20 participants, women were encouraged to choose if they would like to participate in one or both types of interviews. The latter participants all took part in a condensed interview only.

Of the 38 interviews conducted, most interviews (34 interviews) were audio-recorded and transcribed verbatim. Four interviews were conducted by telephone, and the remaining were conducted face-to-face. Interview notes were taken when the interviewer perceived that the recording may be inaudible or when the participant continued to share after the tape recorder was turned off. Four interviews were not recorded as the women did not consent to being recorded, and extensive notes were taken. Hence, a total of 34 interview transcripts, eight interview notes, and two qualitative survey responses were included in the dataset for analysis. All sources were included in the data analysis to ensure that every participant’s voice was represented, regardless of their FDV circumstances, which may have prevented them from recording the interview or completing it face-to-face, online, or via telephone. This responsiveness is aligned with the CBPR principles underpinning the study.

All participating women were also asked to complete a brief written survey to collect demographic information, including their year and country of birth, ethnicity, arrival visa, number of years living in Australia, marital status and children, languages spoken and spoken English proficiency, highest level of education, study area, and employment status. The collection of these data provided context to the experiences the women shared.

### 2.6. Power and Control Wheel

The interview guides were designed to map the CaLD migrant and refugee women’s experiences of FDV within the framework of the Duluth Power and Control Wheel [32]. The Duluth Power and Control Wheel is a model of intimate partner violence developed in 1981 by the Minnesota Domestic Abuse Intervention Project from the experiences of intimate partner violence shared by women in focus groups [32,33]. This project was part of a broader systematic community response to domestic violence, and the model has since been utilized and adapted throughout the world to reflect intimate partner violence in different populations, such as Pacific communities [33], and different settings, such as on social media [34].

**Figure 1:**
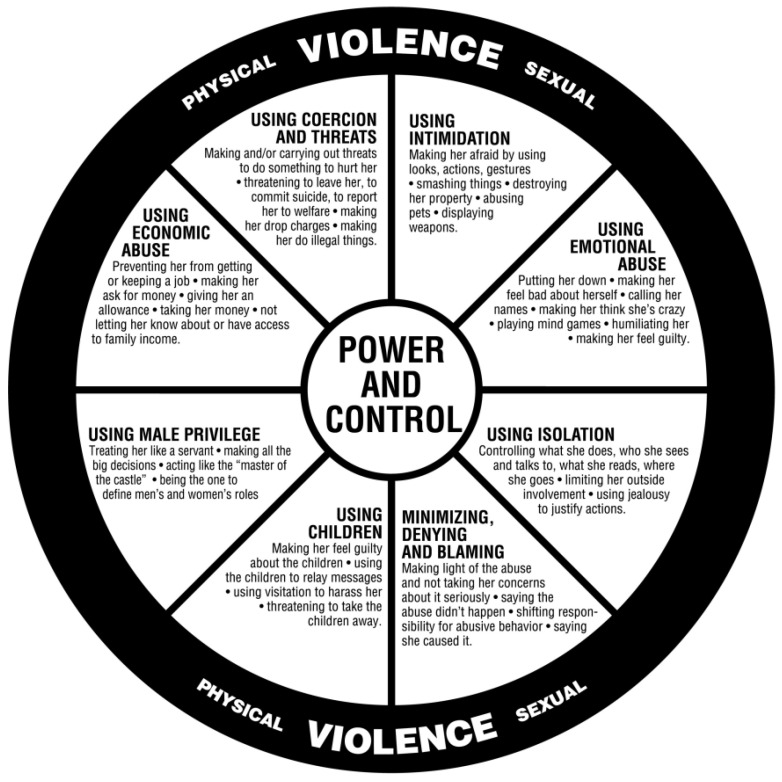
The Duluth Power and Control Wheel [32].

The Duluth Power and Control Wheel [32], pictured in Figure 1, centers power and control in the middle of the wheel, surrounded by eight elements of abuse experienced by women from male perpetrators: using coercion and threats; using intimidation; using emotional abuse; using isolation; minimizing, denying, and blaming; using children; using male privilege; using economic abuse. Physical and sexual violence are shown to reinforce these elements. This model has been used to guide the implementation of interventions globally [32,33].

It must be acknowledged that the Duluth Power and Control Wheel was developed in a small, predominantly ‘white’ North American town [35] and may not be able to account for other cultural contexts [33]. Particularly for migrant women from collective cultures, FDV may impact or be exerted through women’s families and community relationships, which may influence women’s responses to FDV and access to support services. Thus, it is important, when applying the Duluth Power and Control Wheel, to consider the cultural context of the population. Another North American organization, Futures without Violence, adapted the model to reflect the forms of domestic violence that immigrant women may experience [36]. In this model, sexual abuse and using citizenship or residency privilege replaced using male privilege and minimizing, denying, and blaming as elements of the wheel. Physical abuse was depicted as reinforcing the eight elements of abuse.

### 2.7. Data Analysis

**Figure 2:**
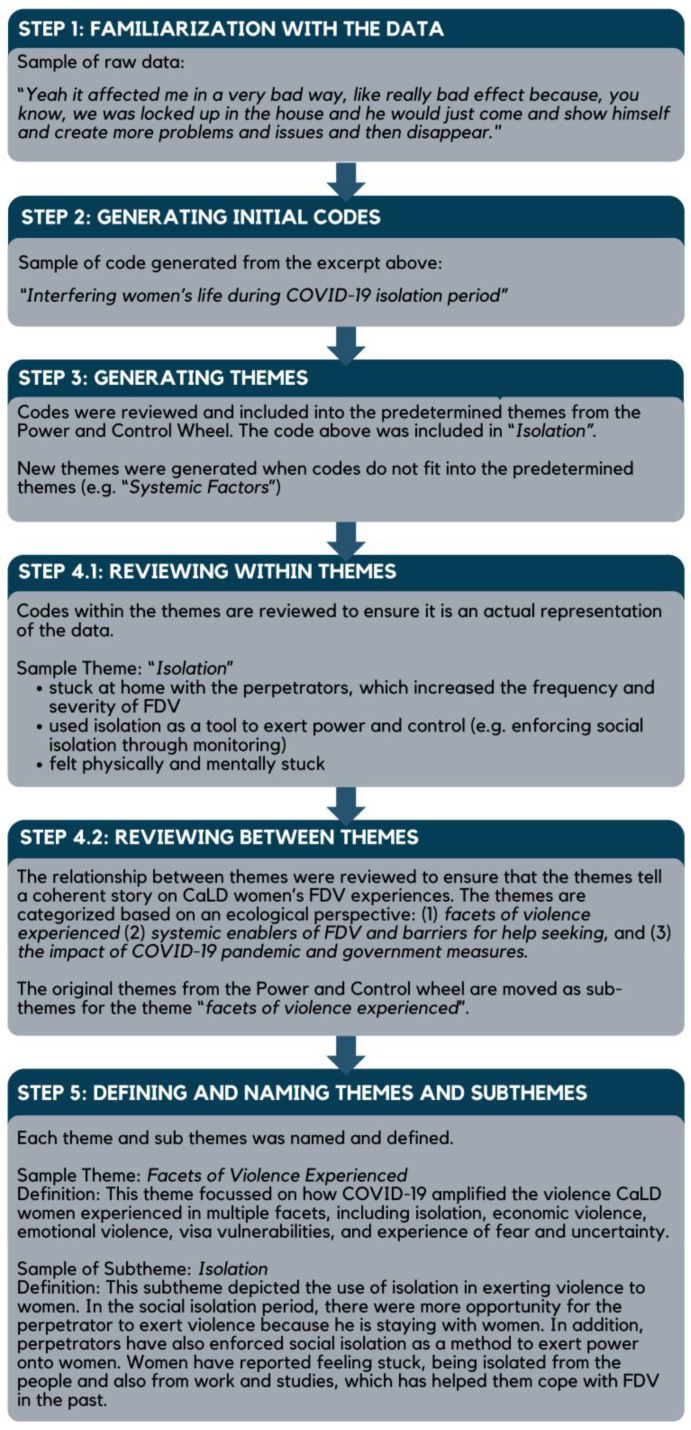
Thematic analysis process with exemplary codes, subthemes, and themes.

Data analysis was conducted on interview transcripts, notes taken during interviews, and qualitative survey responses. A reflexive thematic analysis of the dataset was conducted by the first author (AL) using NVivo 13/R1/2020 software [37] for data management and to facilitate analysis. Reflexive thematic analysis was selected as the analytic approach for its emphasis on reflexivity, facilitating researchers’ critical engagement with their pre-existing biases and assumptions [38]. This analysis was selected as ideal for this research with culturally diverse populations as it encouraged the researchers to examine their own cultural lens. Analysis was guided by the research question, the Power and Control Wheel [32], and underpinned by CBPR principles [23,27]. In alignment with the methodology set out by Braun and Clarke [39], the first author (AL) familiarized herself with the dataset by listening to each interview and reading each interview transcript. Initial codes were generated, listed, described, and compared with one another before being grouped into themes informed by the Power and Control Wheel [32]. A sample of analysis is presented in Figure 2.

### 2.8. Reflexivity

Reflexivity was maintained throughout the research process through collaborative practices to facilitate the researchers’ critical acknowledgement of their subjectivity and how this may have influenced the research [40]. The research team came to this research motivated by a shared dedication to improving the health outcomes and psychosocial wellbeing of CaLD migrant and refugee background women.

The first author, AL, is a CaLD migrant woman born in Southeast Asia with prior experience in supporting others’ mental wellbeing in Australia (e.g., emotional and suicide support services). Her cross-cultural experiences informed her perspectives on the integration of collectivistic and individualistic cultures and how the integration of cultures shaped CaLD women’s understanding and experiences of FDV. She recognizes that her collectivistic cultural lens along with her experiences living in an individualistic society would have influenced the findings generated. GG is a nurse midwife with experience caring for CaLD women experiencing FDV in a hospital setting. These experiences informed her perspectives on systemic barriers to help seeking and service provision faced by CaLD women. She acknowledges that her identity as an English-speaking Caucasian woman born in Australia influences the lens through which she approached this research. HB is a CaLD migrant woman born in Mongolia and brings a unique older person lens to the project as a migrant woman, a mother, and a community member with strong local links in Perth. She conducted most of the interviews with the participants. EM is a young Australian health promotion graduate who assisted with data collection tasks in the project and brings a different lens as a young person to the project. JD is the senior author and SHAKTI project lead since its inception. She is a migrant researcher who has lived and worked in five countries, has led multiple community intervention projects, has a keen understanding of FDV community issues, and strongly believes in knowledge translation.

Throughout data collection and analysis, for example, GG, HB, and AL discussed the experiences shared by interview participants, reflecting on the emotional responses that the participant experiences elicited and the expectations or assumptions that were being challenged. These discussions facilitated an exploration of their personal reflexivity. Methodological and contextual reflexivity were maintained throughout the decision-making process, with the research team taking a particularly considered approach to the power dynamics between the research team and the participating women, such as encouraging the women to select a location for the interview that they identified as safe and familiar and offering opportunities to take breaks or stop the interviews. 

### 2.9. Data Confirmation

To ensure that the data curation was not subjected to personal bias, the first author (AL) met with the interviewers and authors (GG and HB) to discuss preliminary codes and themes as they were being constructed. Preliminary research findings were presented to the CAG to facilitate the contextualization of the findings within local systems and power structures, an important aspect of intersectional research practice into violence against CaLD migrant and refugee women [41].

### 2.10. Ethical Considerations

This study was designed cognizant of the specific ethical considerations of conducting research with people from migrant and refugee backgrounds and in the context of violence against women [25,38]. The research team built on pre-existing community relationships and invested in strengthening the trust between communities and stakeholders [41] by partnering with frontline services and forming the CAG.

At an individual level, as the experiences discussed in each interview included traumatic experiences, participants were provided with a list of local support services, and the services of a counseling psychologist were made available to any participating women who requested additional support. One woman accessed these services. If any women became distressed during an interview, they were encouraged to take breaks and to stop the interview if they wished to. Some interviews were conducted over multiple sittings for this reason. It was considered important that the women’s right to self-determination and their choice to continue the interviews were respected [42].

All women who expressed interest in participating in an interview or qualitative survey were provided with a participant information sheet and encouraged to discuss this with a member of the research team to ensure that they had the opportunity and were comfortable to ask any questions. They were advised multiple times throughout the process that their participation was completely voluntary and would not affect their relationship with the partner agency they were engaged with. All participants signed a written consent form. Ethical approval was provided by the Curtin University Human Research Ethics Committee (HRE2021-0612).

## 3. Results

Twenty-seven women participated in this study. Women were between 30 and 55 years of age. They reported diverse cultural backgrounds, most frequently Pakistani (*n* = 4, 14.8%) and Indian (*n* = 3, 11.1%). Similarly, the most common country of birth was Pakistan (*n* = 6, 22.2%). Less frequent countries included Colombia (*n* = 1, 3.7%), Egypt (*n* = 1, 3.7%), Ireland (*n* = 1, 3.7%), Somalia (*n* = 1, 3.7%), Taiwan (*n* = 1, 3.7%), Uzbekistan (*n* = 1, 3.7%), Indonesia (*n* = 1, 3.7%), Romania (*n* = 1, 3.7%), Mauritius (*n* = 1, 3.7%), Hong Kong (*n* = 1, 3.7%), Malaysia (*n* = 1, 3.7%), Ukraine (*n* = 1, 3.7%), and Sierra Leone (*n* = 1, 3.7%). Most women spoke at least one language other than English (*n* = 23, 85.1%). Most women reported that they spoke English well (*n* = 4, 14.8%) or very well (*n* = 14, 51.9%).

The type of arrival visa and the number of years living in Australia were also diverse. Common arrival visas included partner (*n* = 10, 37%) and student visas (*n* = 5, 18.5%). Almost half of the women had been living in Australia for 10 or more years (*n* = 12, 44.4%). Most women had completed a university degree (*n* = 18, 66.7%), although two women reported never having attended any schooling. Only half of the women (*n* = 13, 48.1%) were engaged in paid employment or study. Women’s familial relationships also varied, with differences in marital status and number of children. Most women were separated (*n* = 13, 48.1%) or divorced (*n* = 7, 25.9%) and had at least one child (*n* = 19, 70.3%). Please see Table 1 for detailed participant characteristics. The unique circumstances of each woman shaped their experiences with FDV.

All participants were facing different circumstances during the COVID-19 pandemic. Ten participants were in a relationship with the perpetrator during the pandemic, of which there were two participants who were separated from the perpetrator due to border closures. Eight participants had left the relationship before or during the pandemic but were still enduring the ongoing impact of FDV in their lives in the midst of the pandemic. Seven participants stated that FDV occurred after the pandemic, of which there were three participants who were separated from the perpetrators due to border closures, and FDV only occurred after they reunited. Two participants had left the abusive relationship many years prior and were no longer living with the ongoing impact of FDV during the pandemic. Three participants did not state their relationship situation during the pandemic. Hence, participants’ experiences during the COVID-19 pandemic were heterogeneous and should not be interpreted as uniform. The length of interviews ranged from 16 min 36 s to 2 h 16 min and 6 s.

### 3.1. Adapting the Power and Control Wheel

**Figure 3:**
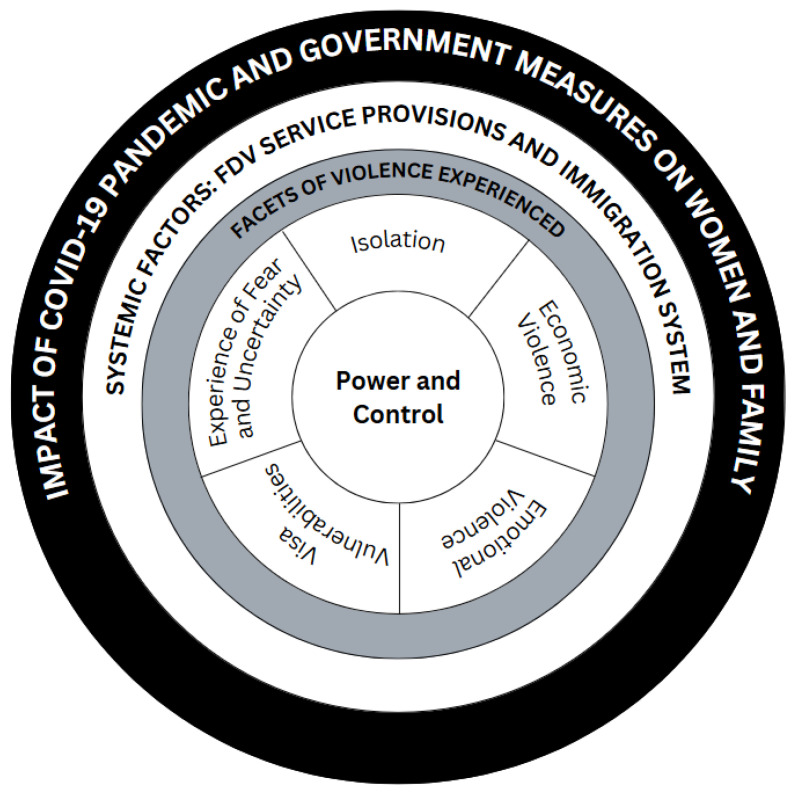
Adapted Power and Control Wheel for CaLD migrant and refugee women in the context of the COVID-19 pandemic.

Three overarching themes were generated from the interview transcripts, interview notes, and qualitative survey responses: (1) facets of violence women experienced based on the Power and Control Wheel, (2) systemic enablers of FDV, and (3) COVID-19 pandemic and government measures’ impacts on women and their family’s lives. An adapted Power and Control Wheel was created to highlight the experiences of CaLD migrant and refugee women in the context of the COVID-19 pandemic, as shown in Figure 3. The adapted Power and Control Wheel has been approved by the Domestic Abuse Intervention Programs, the governing institution for the Duluth Power and Control Wheel.

The adapted Power and Control Wheel highlights how the COVID-19 pandemic impacted CaLD migrant and refugee women who experienced FDV on different levels. The COVID-19 pandemic was used as an opportunity and tool to exert violence on women through different facets of the original and immigrant versions of the Power and Control Wheel. In our adaptation, power and control remained at the center of the wheel, exerted through isolation, economic violence, emotional violence, visa vulnerabilities, and experiences of fear and uncertainty, explored within the first theme, the facets of violence experienced. The next layer of the wheel, explored in the second theme below, shows how systemic factors such as FDV service provision and immigration regulations played a role in enabling FDV. However, these factors acted as barriers for help-seeking behaviors when services were withheld from women in need. Lastly, the COVID-19 pandemic itself impacted many people through different means, which contributed to FDV or enabled women experiencing FDV to regain partial control and to seek help. This is explored in the third theme. A description of themes and subthemes is presented in text below with supporting quotes from interview transcripts. Participants are referred to by an identification number such as P1.

### 3.2. Facets of Violence Women Experienced

This theme depicts how women experienced different facets of power and control and the violence exerted upon them during the COVID-19 pandemic. Not all women experienced every facet. The following subthemes will be explored: (1) isolation, (2) economic violence, (3) emotional violence, (4) visa vulnerabilities, and (5) experiences of fear and uncertainty.

#### 3.2.1. Isolation

This subtheme depicts the use of isolation in exerting violence. Women were isolated at home due to the pandemic restrictions and border closures, which amplified pre-existing violence and mental health strain. For some, the government COVID-19 restriction measures amplified ongoing abuse, as women were locked in the same house as their perpetrator. As one woman stated, “he was torturing us, especially during COVID because he was spending more time at home” (P3). Violence during the restrictions also occurred as women were isolated from those they cared about. P4 stated that she could not talk to her family because the perpetrator was watching her and only allowed her a certain time limit to talk to them. As the women had to stay at home and many were forced to cohabitate with the perpetrator, the perpetrator could monitor her, isolate her from others, and exert violence whenever they wanted.

“Yeah it affected me in a very bad way, like really bad effect because, you know, we was locked up in the house and he would just come and show himself and create more problems and issues and then disappear.”

(P19)

When describing being isolated due to the pandemic, women used the words “prison” (P4), “exhausted and frustrated” (P2), and “lonely” (P4, P5). Many participants identified a change in their own personalities and circumstances since the isolation period. P4 described herself as being more dynamic before COVID-19, and she was going to university and working; however, during the pandemic, she described feeling as though she was overthinking everything and unable to leave the house.

Isolation during COVID-19 was not only in the form of physical isolation, it included social isolation and isolation from work and school, which had previously given women a sense of purpose and an escape from FDV. For example, one woman stated, “I really struggled with the lockdown, because I’m quite a social person. I was studying, so that was sort of my saving grace” (P7). The notion of feeling physically and mentally stuck in the midst of ongoing FDV or separation from the violent relationship was distressing at times for many of the women.

Women also felt isolated during the pandemic as they needed to manage the ongoing impacts of FDV without the support of their family members, who were in another country due to the isolation measures. P13 described a feeling of helplessness when unable to disclose FDV and seek support from her mother in another country, as she did not want to cause more anxiety for her family.

“We still talk on the phone every single day. She’s my best friend, but it got to the point where I stopped ringing her because I was so skinny [due to the violence]. That she would just panic. And by this stage, we were already in lockdown, and I couldn’t leave the country, and they couldn’t come into the country because of COVID. So she knew that there was nothing that she could do, from [my home country], and I knew that there was nothing that she could do. So I just stopped calling because she was just so worried.”

(P13)

Intentionally leaving women in isolation was also one of the ways perpetrators exerted violence on women. Along with enforcing social isolation through constant monitoring, one participant perceived that her in-laws intentionally exerted violence by isolating her when she contracted COVID-19. This participant had health vulnerabilities. She described the following:

“I was away in [my home country] due to the visa issue I couldn’t come and I was stuck with his mother and his mother’s sister at his place back home. So, although it was not mandatory for me [to stay with them], I did it out of goodwill but it kind of turned back on me because I was given COVID by them and they all left me and went to another city…. 21 days of isolation there almost killed me. I used to call my friend and cry daily”.

(P2)

Hence, isolation was a distressing period for women. They had to contend with the COVID-19 pandemic, and the enduring impact of FDV, whilst being stuck at home with the perpetrator without their usual coping mechanisms to support them.

#### 3.2.2. Economic Violence

Women described the violence they encountered in the form of financial control and coercion. In the midst of the societal economic hardships, the perpetrators were described as amplifying the negative outcomes of COVID-19 by withholding financial support. P27 stated that the perpetrator withheld financial support during the pandemic when she was stuck in her home country. The perpetrator did not pay for rent or provide money for food, and she struggled as she was not working. Similarly, P5 needed to borrow money from others and was at risk of homelessness due to the perpetrator withholding financial support from her. P5 described, “he just made my life miserable and he didn’t send me any money, then I had to borrow a lot of money”.

The perpetrators not only exerted violence by withholding money but they also took or threatened to take money from women to exert power and control. P5 stated that she anticipated that he would make her homeless by withdrawing all her money from her bank account. Similarly, P3 stated that her partner wanted to take money awarded to her business through a government subsidy, the JobKeeper Payment, to control her life.

“So COVID came, and, you know how [businesses] had to close down completely. So government been paying us the JobKeeper to keep our businesses. Another fight started there. This money is a lot of money. You should give it to me, I can manage your life. Why are you not letting me manage your life? I said, look, make money, manage your own money and your life. Why are you always want to manage me?”

(P3)

Money was a challenge for women throughout the pandemic due to the combination of the ongoing financial crisis experienced across society and perpetrators’ use of economic violence. Such economic violence not only impacted women’s wellbeing but also created more uncertainty and less security for women as money is essential for survival.

#### 3.2.3. Emotional Violence

This subtheme describes the ways perpetrators put down women emotionally to exert power and control during the COVID-19 pandemic. Women described being put down by perpetrators who disregarded their health and wellbeing, diminished their abilities, and placed blame upon them for the violence exerted.

Women’s health and wellbeing were disregarded and perceived as not important. A participant stated that her in-laws did not allow her to take a COVID-19 test when she displayed symptoms.

“I started having symptoms, and they all were in denial. When I asked them that I need to get my COVID test done, they said that you have lots of money to waste, why do you want to get a COVID test done? Because it costed a lot then.”

(P2)

To the participant, it seemed that her in-laws put her down by disregarding her COVID-19 symptoms and overall health as a way of exerting power over her. The family further exerted violence by diminishing her emotional needs and demanding her to value her in-laws over herself. The woman also described her emotional distress upon experiencing the death of a relative due to COVID-19. Her in-laws also disregarded these emotional circumstances and told her to return to her mother’s house when she contracted COVID-19. Below she describes the situation.

“And when it happened, I just went—I was very, very depressed. And they showed no remorse. They were very weird… they said that, you know, I should go home. Why am I acting out?… So I was very angry at that as well.”

(P2)

Women also received the blame for the abuse and stress the perpetrator encountered during the COVID-19 pandemic. Perpetrators were frequently described as shifting responsibility from themselves to participants and blaming them for the violence exerted upon them. P19 explained that her husband had been “doing the wrong things”; however, he blamed her by stating “you are a bad mother, you are a bad woman” and “the kids are ruined by you”.

By disregarding women’s health and wellbeing, diminishing their abilities, and blaming them for the violence exerted, women described being put down emotionally by perpetrators so that the perpetrators had control over them, showing that they were more dominant and powerful, and leaving the women in an inferior state.

Women’s experiences with emotional violence were also aggravated by being forced to cohabitate with their perpetrators. One woman was locked in her home. She felt that the only thing she did for an entire year was cook and wait for the perpetrator to come home. Though she encountered emotional abuse, she felt unable to leave the relationship due to being emotionally dependent on him.

“And because of the lockdown. I was home every day. There was nothing I could do except wait for him to come home and cook for him. That was all I did for a year…I’ve already developed such deep feelings and dependence on him…I didn’t depend on him financially, but emotionally. Because he was the only thing in your [my] world for a whole year…we had to make up because it felt like if I lost him, I lost my world.”

(P23)

Such experiences further enhanced the emotional violence women encountered. They experienced not only emotional abuse or neglect but also an emotional dependence towards the perpetrator that developed due to the pandemic restrictions. COVID-19 measures allowed women to be put in a position where they were not only physically stuck with the perpetrator but also emotionally, hence making it even more difficult to leave the abusive relationship.

#### 3.2.4. Visa Vulnerabilities

This subtheme explores the ways perpetrators used women’s visa vulnerabilities to exert power and control over them, leaving them in a more vulnerable position. Some means of manipulating visa vulnerabilities included threatening to cancel women’s visas (P14, P25), threatening to contact the government to arrest them (P21), withholding women’s partner visa applications (P2, P26), undermining women’s needs when facing visa vulnerabilities (P21), treating women as inferior due to their visa dependency on the perpetrators (P7, P13), and women choosing to tolerate abuse due to visa dependency (P9).

The impacts of visa vulnerabilities were amplified by the circumstances of the pandemic. P23, whose perpetrator chose not to apply for a partner visa, described the overlapping impacts of visa vulnerabilities and the pandemic.

“I didn’t know if I could stay because of the permanent residency…I was panicking inside every day. You’re anxious and you don’t know what tomorrow’s like. I was on a tourist visa and I only had three months. I had to keep extending it. Who knew what was going to happen—next? And I have asthma, so before—without the vaccination—if COVID came back home, I was led to believe that it would be really severe for me.”

(P23)

P23 described the overlapping and amplified anxieties she faced due to the uncertainties of her visa and the nature of the COVID-19 pandemic.

In different circumstances, perpetrators also made use of their familiarity and well-established life in Australia to exert abuse on women who had newly arrived in Australia. One perpetrator expected the participant to be unable to return to Australia due to the border closure and her visa expiring. However, when she arrived in Australia, the perpetrator used his privilege to withhold support (e.g., basic necessities) from her. Such situations placed women in a vulnerable state, where they were dependent on the perpetrator for access to money, daily supplies, and accommodation.

“[I] don’t have any single thing. I asked him that I left my—some of my sweaters and shawls and everything. Can you please give me one of them? He said, I threw all of your luggage outside. I don’t have any single thing of yours in my room. He already threw it. Because he knew that [I] can’t come in. That’s the pandemic days. No flight will come. And my visa was about to finish.”

(P21)

P21’s vulnerability and stress were further exacerbated by her cultural norms, where she could not seek support from her cousins as they were male.

“I couldn’t stay with my cousins because they all are males…I said to my cousin that-that you can stay inside [the house] and I can like, stay outside in your car, but I can’t stay alone with you all men. Because I was very, very stressed.”

(P21)

Throughout the COVID-19 pandemic, visa vulnerabilities were specifically used as a tool for perpetrators to exert violence against women, and the negative impacts of visa vulnerabilities were further heightened due to the ongoing pandemic restrictions. Systemic factors influencing the use of visa vulnerabilities as means of exerting power and control are explored further in the theme *Systemic Enablers of FDV and Barriers to Help Seeking*.

#### 3.2.5. Experiences of Fear and Uncertainty

In this subtheme, the combined experiences of women as a result of coercion, threats, and intimidation from perpetrators are described. The original Power and Control Wheel described the use of coercion, threats, and intimidation as a way for perpetrators to exert control. However, this theme focused on the common experiences of fear and uncertainty resulting from the perpetrator’s abuse, as well as intersecting fears due to the COVID-19 pandemic.

Women all encountered the fear of contracting COVID-19 during the pandemic. However, this fear was misused as a tool to exert violence on women. One of the threats that perpetrators used to instill fear in women was the risk of contracting COVID-19.

“I was telling him, you’ve been exposed out and he was like, so what and he was coming and grabbing my son and he was kissing top to toe, like, now your mummy get COVID too, like that’s how he was acting.”

(P3)

In this example, the perpetrator’s behaviors were perceived as putting the woman’s and child’s health at risk. P5 also reported her perpetrator threatening her life with the risk of contracting COVID-19 because she had pre-existing health vulnerabilities.

“I thought maybe I wouldn’t survive just because of this… condition, he would… make me die and everyone would say oh she died because of COVID. No one would come to know why I died.”

(P5)

In both instances, perpetrators used the risk of contracting COVID-19 to threaten women. The outcome of these threats was to instill fear to control women.

Another aspect of fear was uncertainty. Though it was not explicitly stated, many women reported feeling fearful about the harm that was to come due to FDV as well as the uncertainties due to the nature of COVID-19.

P3 described that she was always feeling fear and uncertainty. She feared the violence the perpetrator might exert, and unknown threats to come, “I was always in fear. What is the next plan? What is the next plan? That’s why I’m always, whenever he goes quiet, I’m worried that what is the next plan” (P3). This fear, created by uncertainty, was magnified due to being forced to be in the same house as the perpetrator. Besides the woman’s own fear, her child also experienced fear of the perpetrator, “once I remember when my son was with me, he asked, is… daddy to come and see him? And I’ve seen my son had lots of fear in his face” (P3).

The combined effect of the fear of uncertainty associated with COVID-19 and FDV severely impacted women’s wellbeing. P25 described the intersecting fears of COVID-19 and domestic violence (DV), as well as how the fear was exacerbated due to uncertainty.

“I think the fear I had during COVID, it added up more when the DV happened…I was constantly in flight or fight mode. I was constantly scared. What’s gonna happen next? What’s gonna happen? Am I gonna get punched today? Am I gonna get shouted? I’m like that every day now.”

(P25)

This excerpt shows this woman’s fear of COVID-19 and FDV, where she was unsure when the abuse would happen, or what would happen due to the pandemic. Women were constantly living in fearful and anxious states due to these uncertainties.

There were several actions that women took in response to fear. Some reported that they needed to please the perpetrators and do all that they wished to calm them down (P2, P3), while another participant reported that she avoided returning home because she was afraid of the perpetrator (P5). P25 described herself as being hypervigilant and cautious toward people due to the fear of contracting COVID-19 from others, as well as being fearful that others might take advantage of her. She described the following:

“I’m very careful. I don’t trust people. I’m very scared…because of what happened, I just don’t want anybody to come in…If someone comes and says…we [will] help you. I’ll always ask, are you for real? What do you want in return from me? I’m scared. And the police are saying, no, we are here to help. They’re genuine.”

(P25)

Feelings of fear and uncertainty were common experiences for most participants. Fear and uncertainty were used as tools to exert violence over women and were also outcomes of the violence women experienced.

### 3.3. Systemic Enablers of FDV and Barriers to Help Seeking

This theme depicts how systems played a role in enabling FDV and acted as barriers to help seeking. Two perspectives of systemic factors will be discussed: (1) FDV service provision and (2) the immigration system.

#### 3.3.1. FDV Service Provision

Women reported that it was more challenging to navigate services and seek help with the COVID-19 restrictions in place. P16 shared that she struggled to find help as she could not go to the general practitioner or her support person’s house. She also found it hard to go to food banks and obtain food vouchers. Another hindrance in navigating services during the pandemic for P16 was the difficulty in traveling by public transport during the pandemic.

Some services were also perceived to use the pandemic as an excuse to withhold support. P5 described this as a “pandemic effect”. She perceived that these services could have used adaptive measures to ensure safety during the pandemic.

“And many of the organisations taking excuse, or this is COVID time so we can’t do it. We can’t give you appointment, this is delay, pandemic effect was delaying of the things because even it is very safe to talk... they could put mask, sanitiser was there. A lot of things you can take care of while doing but still organisation was making excuse the pandemic.”

(P5)

In short, the COVID-19 pandemic made accessing and navigating help challenging. Moreover, some services were perceived to have withheld support due to the pandemic, which made help seeking even more challenging for women.

#### 3.3.2. Immigration System

Participants’ identities as migrants, governed by visa conditions and location restrictions, played a role in amplifying the violence exerted against them. One participant, P16, stated that she was unable to receive support during the pandemic because of visa conditions, and she found it difficult to adapt to her separation from her perpetrator. She stated that her visa conditions restricted her from studying, working, and renting a room, and she did not have access to social security services, which made navigating life post FDV difficult. P23 described the inability to work due to visa conditions as “enhancing the feeling of worthlessness”.

Alternately, one participant also reported that despite meeting the visa requirements, she was unable to receive government-funded financial support because she was located outside of Australia during the pandemic due to the border closures. She was unable to transfer her money from Australia to her home country due to system procedures (sending remittances overseas). In addition, she was unable to receive financial support from the government, despite being eligible to receive support with her permanent residency, because she was involuntarily residing outside of Australia. She overcame her financial crisis through superannuation withdrawal. P5 stated “even I’m permanent resident, still I have to be [in Australia]... They didn’t understand that I’m been stuck due to COVID, I need money up there [home country], but I had to borrow”.

Another aspect of the immigration system enabling FDV was through women’s perceived systemic barriers. One woman was afraid to reach out for help during the pandemic as she feared this might be used against her permanent residency application.

“I called the suicidal hotline a few times because I just felt so helpless…The first time, I hung up briefly, because back then I wasn’t a permanent residency yet. It came to mind that actually your mental health will impact your ability to obtain a permanent residency. Your visa could get rejected based on severe mental health conditions. So nobody wants to take the risk.”

(P23)

Immigration policies and procedures left women unsupported during the pandemic. Women were placed in a vulnerable position based on their visa status and location of residence, amplifying the negative impacts of FDV and COVID-19.

### 3.4. Impact of COVID-19 Pandemic and Government Measures on Women and Families

This theme describes the impact of the COVID-19 pandemic and government measures on women and their families, which indirectly enabled FDV or reduced the impact of FDV. Some also described that the COVID-19 pandemic did not have much impact on their lives due to the ongoing effects of the FDV that they were experiencing.

#### 3.4.1. COVID-19 Pandemic Enabling FDV

On a personal level, both women and the perpetrators experienced individual stress from the pandemic. The mother of one perpetrator died during the COVID-19 pandemic, yet he was unable to attend the funeral due to the lockdown restrictions. The participant attributed this event to her husband recommencing his use of marijuana, which later escalated to FDV due to the impact of the drug.

“His mum passed away during the COVID time. And he couldn’t go back to [home country]. He seems very—[Interviewer: Upset with that.] Yeah. Upset with that. And then when he has a delusion—he taking the marijuana, and then he keeps saying his mom’s still alive, and we trying to cheat him, say his mom passed away. He opened all the windows and doors, just say, oh my mom is coming, something like that…I think if at that time my husband was able to visit his families in overseas, the things may be a little bit different.”

(P11)

The COVID-19 pandemic did not solely impact participants on an individual level but also impacted them on a familial level. P7 iterated “I could kind of see the family falling apart during COVID.... It was just such a lot going on within—within the house with each individual person”. P7 herself experienced individual stress from being isolated. In addition, at a familial level, her partner had work pressures, her daughter was struggling in school, and the family was struggling to buy masks to protect themselves during the pandemic. The multifaceted stress that the individual and the family went through during the pandemic impacted the family functioning and marriage, ending in marriage dissolution.

“He was at breaking point with work and I think that contributed to us separating because his workload tripled. So where most people were laid off, and he was working from home, I’m studying from home, the kids were off school. I was still expected to do everything I did while he was just working all the time on his computer seven o’clock till seven…and I think that was a big contribution to us separating. It was a huge stress for us… He was under an enormous amount of pressure and something had to give and unfortunately it was me.”

(P7)

#### 3.4.2. COVID-19 Pandemic Reducing the Impact of FDV

Despite the exacerbation of FDV for most women, some reported that the pandemic and government measures were in their favor as they isolated them from the harms of FDV. Some stated that the isolation measures actually led them to separate from their perpetrators, enabling them to feel safer. P10 shared that she was able to go out freely and safely due to the isolation measures. She did not feel afraid because she perceived that there was no one outside to hurt her or offer her drugs. However, P10 acknowledged that this was only possible because the perpetrator was no longer living with her during the pandemic.

Another participant stated that the isolation measures were helpful for her as she could be aware of her husband’s whereabouts.

“I remember that it was actually quite positive because when all of the bars shut, it meant that he couldn’t go out drinking… So he stayed at home. You know, so at least I knew where he was. So that some of the lockdowns actually benefited me because he couldn’t go missing for five days.”

(P13)

For both these participants, isolation measures perhaps gave them some control and certainty in their lives, which was not previously evident in their FDV experiences, hence reducing the intensity and ongoing effects of FDV.

Though some women reported that the lockdown measures removed their usual escapes from FDV, such as work and school (see the subtheme *Isolation*), some women stated that the isolation measures helped distract them from the ongoing impact of FDV, “thinking of COVID helped me not think about the police and VROs [Violence Restraining Orders]. It was a distraction.” (P22). This participant further described it as “a blessing in disguise”.

Some participants also stated that the COVID-19 pandemic was a period of reflection that helped them pivot their priorities in life. P1 shared that she took COVID-19 very positively as she gained clarity that her children were the priority and that she needed to leave the FDV relationship for her children’s best interests.

“And I would take that in a very positive way because I think it was a point where we all realized that, okay, family’s more important… So I think COVID has given us a lot of eye-opening points as well in our lives where we realised that—what is the priority and what’s not.”

(P1)

The quarantine system was also helpful for one participant who realized what she experienced was FDV through interacting with others, and FDV information provided by the healthcare team in the quarantine facility: “there was a good thing in quarantine, health team... were calling for your wellbeing [everyday]” (P5). Some participants also stated that they were supported by the government during the pandemic. “They put my claim very immediately. And they said to me that just go to the [bank] and get [money]. That was my first payment which I got from the government” (P21).

Another aspect of this subtheme was the impact of the COVID-19 pandemic on reducing the mental health impact of FDV through collective suffering. Women no longer felt alone, as they were aware that many people worldwide were also suffering, “my life was going into chaos, but COVID was a little bit weird because I was happy. I was happy because I was not the only person whose life was chaotic” (P22). The notion of having a shared experience with many women going through similar hardships was a comfort for P22, knowing that she was not alone in her experiences. Similarly, P14 also shared that she felt better when she was isolated with other women in a refuge during the pandemic. As she described, “this [was] better because [I was] isolated with other women in the same situation [and] condition” (P14).

#### 3.4.3. COVID-19 Pandemic Paled into Insignificance

Some women stated that the pandemic and government measures had little impact on their lives due to the intensity of the stress they were going through in the FDV relationship. As they were affected by the impact of FDV, the pandemic was no longer a point of concern for them. When asked how the pandemic had affected her experience of FDV, P15 stated,

“Well there’s nothing specific for me, during the pandemic, it’s still the same, you know, as it was before pandemic or after pandemic. It’s just him. There is no other problem in my life. It’s just my ex is the biggest problem.”

(P15)

P7 had similar thoughts regarding the insignificance of the COVID-19 pandemic due to dealing with the ongoing effects of FDV. P7 stated, “by then [partner] had left and I was dealing with other things, so the pandemic sort of paled into insignificance really”. Hence, it is evident that the impact of FDV could be greater than that of the pandemic, which was consequently perceived as insignificant or not of priority for some women.

## 4. Discussion

### 4.1. Summary of Findings

This paper explored CaLD migrant and refugee women’s experiences of FDV in the context of the COVID-19 pandemic in Western Australia. On an individual level, women experienced various facets of violence during the COVID-19 pandemic: isolation, economic violence, emotional violence, visa vulnerabilities, and the experience of fear and uncertainty. Apart from the violence exerted by perpetrators, women also experienced greater challenges in accessing and navigating services during the pandemic, which hindered them from accessing and receiving help from service providers. Systemic policies, especially where visa and geographical location were made a pre-requisite for receiving help, were identified by women as a barrier to support and exacerbated the violence they experienced from FDV. Lastly, the COVID-19 pandemic and government measures had different impacts on women and their families. Some women benefited from COVID-19 measures as they regained some power and control over aspects of their lives or had the opportunity to seek help, while for some, the COVID-19 pandemic impacted the perpetrator and family functioning, which contributed to or led to FDV. For some, the COVID-19 pandemic was not a main priority or contributing factor as they were already occupied with the ongoing impacts of FDV.

### 4.2. The Experience of Violence During the COVID-19 Pandemic from CaLD Women’s Perspectives

The rise in FDV globally during the pandemic has warranted research to explore this issue and urged for actions to be taken [43]. Usher and colleagues described the pandemic as the ‘perfect storm’ for domestic violence [44]. Consistent with our study results, many women experienced violence where the perpetrator used the COVID-19 virus and the existing social distancing measures to exert abuse and control [45,46,47]. This included using the risk of contracting COVID-19 to threaten women and enforcing social isolation to prevent women from seeking help. Many women were also forced to cohabitate with the perpetrator, which increased the frequency and intensity of the emotional, physical, and sexual abuse experienced [45,46,47,48]. Additionally, other studies also described the enormous stress most families endured during the pandemic, which includes anticipated fear and uncertainty associated with the pandemic, financial stress and employment uncertainty, and parenting stress to care for home-schooled children [49,50]. The mental health strain within the family was found to increase outbursts of anger, which heightened the risk of FDV. Though these international and Australian studies did not focus on migrant and refugee communities, it is evident that our study participants with migrant or refugee backgrounds experienced similar violence and hardships.

Though the CaLD migrant and refugee population experienced similar violence, they were more vulnerable in their FDV experience during the pandemic in Australia, with an increase in frequency, intensity, and complexity [51,52,53,54]. One of the most prominent stressors for women was the power differences between males and females. Our study found that the perpetrator and their family set many gendered expectations, and they used this presumed power to exert control over women in all facets of their lives. To illustrate, women were expected to give the perpetrator their money as this was known as the perpetrator’s presumed right. This is common in certain communities that have set cultural expectations and norms where men are viewed as the head of the family, and women are expected to take on the supporting role to care for the family [55]. 

These power differences not only increased the risk of abuse, but it was also manifested in women’s perceived responsibility to tolerate violence, which also encouraged FDV and hindered help-seeking behaviors. We found that CaLD migrant and refugee women were often the ones being blamed when the perpetrator or family was struggling during the COVID-19 pandemic and were on the receiving end of emotional frustration and stress. Our findings were consistent with a recent integrative review that explored the impact of sociocultural norms on women’s decision to disclose intimate partner violence globally [55]. They found that sociocultural norms such as family honor, gender roles, and stigma in their culture shaped women’s experiences and decisions, and these were common experiences across several collectivistic countries and ethnicities that adopt familial collectivistic values. This collectivistic familial expectation encouraged CaLD women to be silent, sacrifice their health and wellbeing, and tolerate the abuse as a result of their gender expectations and to preserve family cohesion and honor [56,57,58].

Another significant facet of violence that CaLD migrant and refugee women identified in this study was social isolation. Many women stated that they were isolated from their families due to border closures, which were one of the prominent features of the COVID-19 pandemic in WA. For some, these border closures were even triggers for FDV in their family. Studies from the United States and Australia have found that migrants and refugees were more prone to social isolation during the pandemic as they might have faced settlement-related challenges such as language skills, fear of racism, intimidation by mainstream society, and not having family and friends in the same country to support them [58,59]. Social isolation can be exacerbated when being locked with their partner due to the ongoing pandemic restrictions [59]. This vulnerability has greater significance in CaLD communities as they tend to rely on informal support for FDV due to the fear and mistrust of formal services and to preserve their family honor [60]. During the pandemic, women were no longer able to freely seek informal support due to additional monitoring from the perpetrator [61] or the fear of infecting others with COVID-19 [59].

Lastly, the greatest vulnerabilities that were unique to the CaLD migrant and refugee population were visa vulnerabilities. Our study results were consistent with research where visa vulnerabilities not only manifested in the form of perpetrator tactics but also acted as a barrier to receiving support from services. Perpetrators used migration-related controlling behaviors to further exert abuse on women [48]. Some examples of migration-related controlling behaviors from the literature include threatening to withdraw sponsorship, having their dependent deported, or preventing other family members from obtaining visas or entering Australia [48]. In our study, visa vulnerabilities manifested differently, where the perpetrators chose not to help women apply for a partner visa, left women in fear of uncertainty during the pandemic, or amplified the settlement challenges of being a new migrant to exert abuse on women.

In addition to migration-related abuse, CaLD migrant and refugee women also faced further hindrances in receiving support or navigating hardships during the pandemic because of their visa status. Australian studies have concluded that temporary visa holders are marginalized, being ineligible for services, job opportunities, and accommodation through government policies, which puts victim-survivors in a vulnerable position when seeking help [48,62]. They have no guarantee of financial support, and some are ineligible for certain social services. For example, in Australia, individuals on student visas cannot access government subsidies for healthcare through Medicare, an Australian universal health insurance scheme that guarantees Australians access to health services at low to no cost [47,63]. The participants experienced the vulnerabilities of their visas in seeking support during the pandemic. Our study also explored various system provisions that withheld support not only due to visa restrictions but also due to geographical constraints, such as being stuck in another country due to the pandemic, which created barriers for women seeking help. Research has found that these negative experiences with services further amplified the fear and distrust that the migrant and refugee population has with formal support services [64].

Prior research has offered unique insights into specific facets of violence that CaLD women may face, for instance, migration-related violence. However, our findings highlight the interplay between cultural values and expectations and visa status and systematic policies during the COVID-19 pandemic that have placed CaLD migrant and refugee women in a vulnerable situation. As researchers, we find it difficult to use themes to categorize the experiences that women faced during the COVID-19 pandemic and amid FDV, as it is impossible to sectionalize these experiences. The intersectionality of the violence experienced by women goes beyond what is presented in this paper. The themes generated can only provide a brief understanding of the facets of suffering women encountered.

### 4.3. The Experience of Violence Post COVID-19 Pandemic

Our study focused on the impact of FDV during the pandemic on migrant and refugee women living in WA. However, it is important to also consider the changes to policy and practice that have occurred due to the pandemic. The underlying drivers of violence against women were exacerbated during the pandemic, with increased social isolation, lockdowns in WA, decreased access to support services, job losses, financial pressures, and psychological distress being some of the major contributing factors [65]. However, the full extent of FDV experienced during the pandemic remains difficult to fully measure due to the nature of the violence often occurring behind closed doors and the low levels of reporting [65]. The pandemic revealed significant disparities in funding, service availability, collaboration, and outcomes, particularly for CaLD women experiencing FDV [66]. In response to the increase in FDV, a variety of policy and practice initiatives were introduced during and after the pandemic, such as expanded online support services, enhanced collaboration between governments, organizations, and service providers, an increase in the FDV workforce, and additional funding for women experiencing FDV and for FDV services [66]. Moving forward, it is crucial to consider the long-term and ongoing impacts of FDV, as it remains one of the most prevalent global social issues impacting women specifically [67]. Victim-survivors still remain highly vulnerable following the pandemic due to many of the underlying drivers of violence enduring, such as access to employment, housing, income, and support services remaining limited [68].

### 4.4. Experiences of CaLD Women in WA in Comparison to Other Countries

Our study results were similar to other studies that explored the FDV experiences of migrant and refugee women from other countries globally. For example, a study from Portugal found that immigrant women experienced increased types and intensities of FDV and highlighted barriers in help seeking such as cultural barriers, isolation, lack of knowledge, economic dependency, and fear of legal repercussions [69]. Similarly, a qualitative study from the United States also found similar impacts of FDV and COVID-19 on immigrants, for instance, an increased frequency and severity of FDV and increased stress due to economic disruption and familial burden, which contributed to poorer mental health outcomes [70]. Another study conducted in Italy highlighted how the COVID-19 pandemic “converged with sexism, racism, and xenophobia in Italy, leading to increased public and domestic violence against refugee and migrant women.” [71] The study also revealed that many refugee and migrant women in Italy in relationships with Italian men during the pandemic faced ongoing psychological abuse and were unable to access FDV support services due to the influence of existing power dynamics [71]. The study participants identified increased socioeconomic insecurity as a major issue during the pandemic, with significant consequences, including the inability to afford food, support their families, secure safe housing in good neighborhoods, and access healthcare and legal services [71]. Similar experiences were found among migrant women in India during COVID-19, with a 2021 study highlighting that the narratives of women were centered around a loss of livelihood, home, savings, and hope [72]. As outlined, the experiences of CaLD women during COVID-19 were similar across various countries and contexts worldwide due to the creation of new and the exacerbation of existing vulnerabilities among refugee and migrant women.

### 4.5. Implications and Recommendations

In short, CaLD migrant and refugee women have faced many unique challenges that shaped their FDV experiences during the COVID-19 pandemic. Though border restrictions have eased, and the pandemic is no longer a main concern, the impact of FDV experiences for CaLD communities remains an important priority. The Australian Prime Minister declared violence against women a ‘national crisis’ and the prevention of FDV an ongoing national priority on 28th April 2024 [73]. Researchers have voiced that the COVID-19 pandemic was only the trigger that exposed and intensified existing gender inequalities and migrant vulnerabilities [48,74]. The lessons learned from the COVID-19 pandemic around establishing adaptive support and regulations to support the migrant and refugee population need to be taken into account to avoid the worsening of societal and economic burdens of FDV as well as the marginalization of vulnerable groups [73,75].

Women have voiced their recommendations to help them in a time of need, and it is important to implement these at the service delivery, policy, program, and political level. Women voiced a need, desire, and recommendation for person-centered and user-friendly support during the COVID-19 pandemic. Person-centered support not only entails considering their cultural identity (i.e., the nuances of gender roles and concepts of honor and shame) but also the vulnerabilities they have as a migrant or refugee (i.e., visa vulnerabilities). CaLD migrant and refugee women were already vulnerable due to the nature of the COVID-19 pandemic and systematic inequality; our participants voiced that service providers should not have exerted additional barriers when providing support. Service providers could adapt their methods, such as wearing masks and having hand sanitizers, to ensure safety from the pandemic when providing support, instead of canceling their appointments. However, it is important to note the struggles that service providers faced during the COVID-19 pandemic. As FDV cases rose during the pandemic, many FDV providers were facing burnout due to short-staffing, increased workload, and a lack of support [76]. Staff also encountered a high risk of vicarious trauma during the time of the pandemic [76]. Hence, additional funding for FDV service providers is needed to relieve the workload of staff, improve safety protocols, and add wellbeing initiatives in order for staff to provide timely and quality services to CaLD migrant and refugee women [76,77]. In addition to increased funding, provided funding needs to be used effectively and user centered. More tailored and streamlined services should be provided to women to ensure that women receive holistic and quality support.

Women not only voiced the need for user-centered care in FDV services but also in other facets such as workplaces, schools, and families. As the women stated, many families went through financial and isolation hardships during the COVID-19 pandemic, which might have triggered the escalation of FDV. The Australian Institute of Health and Welfare [16] summarized factors from personal (e.g., high psychological distress), relationship (e.g., interpersonal relationships strain with family and friends), community (e.g., school or workplace environment), and societal (e.g., economic crisis, government policies) levels, all contributing to the risk of FDV. These factors were all exacerbated during the COVID-19 pandemic. This highlights the need for preparation and planning for future pandemics, as this support should have been in place during the COVID-19 pandemic to ensure all individuals in the family could have been well supported. Systemic changes are required at all levels (personal, relationship, community, and societal), for instance, embedding healthy work cultures (e.g., reasonable working hours), providing sufficient mental health support (e.g., for parents as well as children), and facilitating pandemic safety (e.g., mask availability). Though all these support systems may not directly relate to FDV, the indirect impact will help to alleviate the stress and tension within the family, which in turn reduces the risk of FDV occurring.

Lastly, women urged for policy change in existing systems that promoted conditional support constrained by visa status and geographical location. Migrants and refugees are often overlooked by the existing support services due to their visa status. People with partner visas, or who are secondary applicants of the perpetrators, are now eligible to apply to stay in Australia on a permanent visa through the family violence provision scheme by the Australian Government if they meet certain criteria [78]. However, half of our participants arrived in Australia on different visas as the perpetrator never supported their visa applications. They were disadvantaged in receiving support and had to bear the fear of uncertainty due to their visa status. We recommend providing family violence provisions to not only partner visas or secondary applicants but to all visa applicants. In addition, given that some women were coerced with misinformation about their visa restrictions, information about FDV and family violence provisions needs to be communicated in a linguistically and culturally safe way to all entrants to Australia. Further research is needed into the best way to disseminate information about FDV and family violence provisions to CaLD people who have migrated to Australia. Whilst the Australian Government announced a number of initiatives to support family and domestic violence initiatives in 2024, these do not provide adequate support or sustained funding [79].

### 4.6. Strengths, Limitations, and Future Research Directions

This study offers nuanced insights into the FDV experiences of CaLD women in the context of the COVID-19 pandemic in WA using a pre-existing framework to address FDV, the Power and Control Wheel [32]. The use of CBPR methodology is a strength of the research design. Using CBPR principles, the voices of the women who shared their experiences were centered throughout the findings and recommendations and will inform the co-design of a frontline response to FDV. Through the adaptation of the Power and Control Wheel, we have developed a resource to assist FDV service providers in mapping the FDV experiences of CaLD migrant and refugee women, facilitating in the provision of timely and effective support for this population. This focus on action in partnership with key stakeholders is a core strength of CBPR enacted in this study.

One limitation of this research is that the impact of COVID-19 may have been greater than that captured in this paper. This may be because women shared their FDV experience without explicitly identifying the impact of COVID-19 pandemic measures like lockdowns, isolation, and a lack of ability to travel, rather focusing on the FDV. This is reflected in the subtheme, COVID-19 pandemic paled into insignificance. It is also acknowledged that women were recruited through partner agencies, which supported women in their FDV experiences. Hence, women included in this study had attained some help and support from community services. The FDV experiences of CaLD migrant and refugee women who had not accessed help were not explored in this paper. The Australian Bureau of Statistics Personal Safety Survey indicated that people do not often seek support for FDV (only 55% of women sought support when they experienced FDV from a current partner) [80]. In addition, individuals often utilize informal supports such as family and friends [80] instead of formal support such as our recruiting community services. Hence, this paper only explored a sub-section of people who have experienced FDV and sought help. In addition, as recruitment was conducted via partner agencies, there is a possibility of some bias based on the nature of the clients who utilized the services, which includes but is not limited to ethnicity and cultural background, age, and geographical location. There is also the likelihood of being unable to recruit participants with complex experiences due to safety concerns. Our sampling approach was necessarily influenced by a focus on prioritizing women’s safety, and the research team chose to honor this by working with partner organizations to recruit eligible participants. By providing an in-depth description of our recruitment approach and the characteristics of the study participants, we have sought to enhance the transferability of the findings and provide an audit trial. Further research into the experiences of migrant and refugee women with FDV who have not engaged with any formal services or support is recommended to identify how their needs may be met.

The participants were from diverse cultural backgrounds and countries of birth, with migrant and refugee women from 17 countries of birth and up to 19 ethnicities. Women were also from various social classes and visa statuses. The diversity of this sample is indicative of the diversity of CaLD migrant and refugee women accessing services in WA. The focus of this study was not the influence of specific factors on women’s experiences such as country of birth, ethnicity, language, social class, and legality. Future research may explore how women’s FDV experiences may be shaped by these factors. However, our study highlighted that there is not one single type of CaLD woman who experiences FDV; i.e., we cannot assume that only women with limited education or women from a specific culture or who have arrived on a specific visa are the only women at risk or who require support.

Finally, the participants in this study described FDV perpetrated by a male intimate partner or former intimate partner in the context of heteronormative relationships. The research team acknowledges that the CaLD community is diverse, and the experiences of CaLD women in LGBTIQA+ relationships are not represented here. This is another area of further research recommended by the authors/research team. In consideration of these limitations, the authors acknowledge that the experiences in this paper are not exclusive, and the breadth and intensity of the gendered impacts of the COVID-19 pandemic are not limited to the findings generated here.

## 5. Conclusions

Our study explored CaLD migrant and refugee women’s FDV experiences during the COVID-19 pandemic. CaLD women not only faced the risk of FDV that was common to non-CaLD women but also the unique facets of violence that exacerbated existing violence or heightened the risk of violence. We recommend that tailored support, not only in the FDV space but also more broadly in the workplace, at the family, political, and society level, be provided to women. Structural inequalities that restrict received support need to be reviewed and changed to ensure all CaLD migrant and refugee women can receive the support they need during the challenging time of responding to the FDV they and their children face.

## Figures and Tables

**Table 1:** Participant characteristics.

	*n* (%)
Country of birth	
Pakistan	6 (22.2)
China	2 (7.4)
India	2 (7.4)
Iran	2 (7.4)
Other	13 (48.1)
Missing	2 (7.4)
Arrival visa	
Partner visa	10 (37)
Student visa	5 (18.5)
Working Holiday visa	2 (7.4)
Visitor visa	2 (7.4)
Humanitarian visa	1 (3.7)
Skilled Independent visa	1 (3.7)
Other	5 (18.5)
Does not know	1 (3.7)
Number of years in Australia	
1–2 years	6 (22.2)
3–5 years	4 (14.8)
6–9 years	5 (18.5)
10 or more years	13 (44.4)
Marital status	
Married	3 (11.1)
Separated	13 (48.1)
Divorced	7 (25.9)
Widowed	1 (3.7)
Never married	2 (7.4)
Missing	1 (3.7)
Number of children	
None	8 (29.6)
One	10 (37)
Two	7 (25.9)
Three or more	2 (7.4)

## Data Availability

The data from the Shakti Project are not publicly available to protect the anonymity of the study participants.
